# Effective substitution of ferrous sulfate with iron oxide nanoparticles enhances growth, antioxidant activity, and stevioside accumulation in micro-propagated *Stevia* rebaudiana

**DOI:** 10.3389/fpls.2025.1569613

**Published:** 2025-04-25

**Authors:** Sher Muhammad, Abid Ali Khan, Muhammad Rameez Khan, Sidra Mukhtar, Abeer Kazmi, Amir Ali, Ayesha Siddiqa, Kayley Aileen Hernández Ramírez, Juan Pedro Luna-Arias, Gabriela Medina-Pérez, Armando Pelaez-Acero, Silvia Armenta, Ajaz Ahmad

**Affiliations:** ^1^ Biotechnology Laboratory, Agricultural Research Institute (ARI) Tarnab Peshawar, Peshawar, Pakistan; ^2^ Department of Chemical Sciences, University of Laki Marwat, Khyber Pakhtunkhwa, Pakistan; ^3^ The State Key Laboratory of Freshwater Ecology and Biotechnology, Institute of Hydrobiology, Chinese Academy of Sciences, Wuhan, Hubei, China; ^4^ The Key Laboratory of Aquatic Biodiversity and Conservation of Chinese Academy of Sciences, Institute of Hydrobiology, Chinese Academy of Sciences, Wuhan, Hubei, China; ^5^ Department of Botany, PMAS Arid Agriculture University, Rawalpindi, Pakistan; ^6^ Department of Cell Biology, and Nanoscience and Nanotechnology Ph.D. Program, Center for Research and Advanced Studies of the National Polytechnic Institute (CINVESTAV), Mexico City, Mexico; ^7^ Institute of Agricultural Sciences, Autonomous University of the State of Hidalgo, Hidalgo, Mexico; ^8^ Department of Clinical Pharmacy, College of Pharmacy, King Saud University, Riyadh, Saudi Arabia

**Keywords:** Moringa oleifera, Stevia Rebaudiana, nanotechnology, iron oxide nanoparticles, secondary metabolites

## Abstract

Nanotechnology, particularly the use of iron oxide nanoparticles (IONPs), has gained significant attention in agricultural research due to its potential to enhance plant growth, development, and stress tolerance. However, the green synthesis of IONPs using plant extracts remains underexplored, especially in the context of agricultural applications. In this study, the green synthesis of IONPs using *Moringa oleifera* leaf extract is reported, with the extract serving as both a reducing and capping agent. The synthesized nanoparticles were characterized using scanning electron microscopy (SEM) and energy-dispersive X-ray spectroscopy (EDX), revealing spherical and polygonal shapes with an iron peak at 6.5-7.5 keV, consistent with the expected size and composition. These IONPs were incorporated into Murashige and Skoog (MS) medium to replace the conventional iron source and evaluate their effects on *Stevia rebaudiana* micropropagation. The results demonstrate that IONPs at lower concentrations (5.60 mg/L) significantly promoted early shoot and root initiation (5.2 and 5.3 days, respectively), while higher concentrations (11.20 mg/L and 22.40 mg/L) delayed growth initiation and inhibited development. Notably, 22.4 mg/L IONPs enhanced leaf growth (length: 3.20 cm, width: 1.90 cm), fresh weight (238.90 mg), and dry weight (20.67 mg), outperforming the positive control (FeSO_4_·7H_2_O). IONPs also increased the total phenolic content (TPC) and total flavonoid content (TFC) in plant tissues, with the highest values (4.54 mg GAE/g and 2.07 mg QAE/g) observed at 22.40 mg/L. The antioxidant capacity, measured by DPPH scavenging activity, was significantly enhanced, reaching 89.70%. Additionally, IONPs promoted the accumulation of diterpene glycosides, including stevioside (4.30 mg/g DW) and rebaudioside A (6.70 mg/g DW), especially at higher concentrations. These findings suggest that IONPs, particularly at 22.40 mg/L, are a promising and environmentally friendly alternative to traditional iron sources, offering enhanced plant growth, improved antioxidant defenses, and increased production of valuable secondary metabolites in *S. rebaudiana*.

## Graphical Abstract

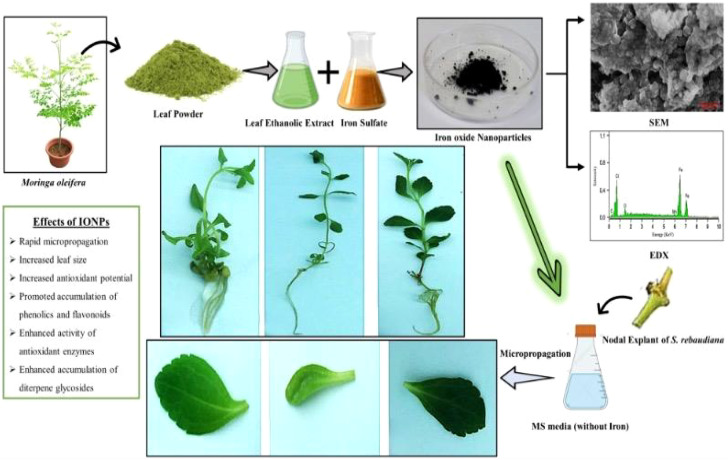

## Introduction

*Stevia rebaudiana* Bertoni, an Asteraceae family member, is famous for producing sweet diterpene glycosides (stevioside and rebaudioside A) (de Andrade et al., 2024). These compounds are non-toxic, non-mutagenic, and 200-300 times sweeter than sucrose (Lankarani et al., 2024). Stevia is widely used as a sugar substitute in the food and pharmaceutical industries because it is calorie-free and has no known adverse health effects. It is commonly used to sweeten soft drinks, confectionery, chewing gum, chocolates, baked goods, jams, and even certain medicines (Huang et al., 2024; Kazmi et al., 2019b). Despite its potential, large-scale production of stevia confronts challenges such as low seed viability and labour-intensive propagation by stem cuttings (Kazmi et al., 2019a). Additionally, the lack of established techniques for growing plants with optimal concentrations of steviol glycosides prevents it from being widely used, which leads to an imbalance between supply and demand in the market. To deal with these challenges, researchers are increasingly focussing on biotechnological approaches to enhance the production of stevia farming (Mahajan et al., 2024; Rukh et al., 2023). Among them, nanotechnology has emerged as an appealing option for agricultural practices. Nanomaterials have the potential to improve plant growth and production, offering an innovative solution to agricultural challenges (Shereen et al., 2024; Parveen et al., 2023; Ahmad et al., 2022; Ehsan et al., 2022). Over the past decade, the application of nanoparticles (NPs) in agriculture has demonstrated significant potential for enhancing crop yield and performance. Depending on their size, shape, and concentration, NPs can exert positive, negative, or neutral effects on plant physiology when absorbed through the roots or leaves (Thiruvengadam et al., 2024; Dey and Nandy, 2024).

In plant tissue culture, elicitation is a potential approach for increasing secondary metabolite production by altering growth conditions and adding particular elicitors (Anum et al., 2021; Murthy et al., 2024; Ghazal et al., 2024). While several studies have investigated the effects of conventional elicitors, such as salicylic acid and jasmonates, on the biosynthesis of steviol glycosides in Stevia, research on the effects of nanoparticles (NPs) on plant micropropagation and steviol glycoside production remains limited (Tavakoli et al., 2019; Moharramnejad et al., 2019; Alsafawi and Şahin, 2024). Research indicates that the effect of metal nanoparticles on Stevia varies with concentration (Ramezani et al., 2019; Khan et al., 2020; Lankarani et al., 2024). At low NP concentrations, plant growth is often stimulated, steviol glycoside synthesis is enhanced, and antioxidant activity is improved (Khan et al., 2020; Sichanova et al., 2022). However, at higher concentrations, NPs can induce phytotoxicity, reducing biomass and limiting metabolite accumulation (Biswas and Pal, 2024; Mathur et al., 2023).

*In vitro* studies have revealed that the specific effects of NPs on plant growth and metabolite production differ significantly depending on the type of NP employed, its concentration, and the environmental conditions under which the plants are propagated (Javed et al., 2022; Iqbal et al., 2022; Ali et al., 2019, 2023; Pathak et al., 2023). Silver nanoparticles, in particular, have been extensively investigated for their impact on plant growth and metabolite production in tissue cultures (Ali et al., 2019; Chung et al., 2018; Gupta et al., 2018; Natarajan et al., 2024; Ali Naghizadeh et al., 2024). Silicon nanoparticles have been shown to promote growth and secondary metabolites production in *Mentha pulegium* L. tissue culture (El-Naggar and Osman, 2024). *Moringa oleifera*, a member of the Moringaceae family, a small tree native to the sub-Himalayan regions of North West India, now found in many parts of the world, including Islands and South America. This plant has been a staple in local diets and holds significant cultural and medicinal value Known as the “miracle tree” due to its reputed health benefits. *Moringa oleifera* has been used for centuries to treat a wide range of ailments, including asthma, bronchitis, chest congestion, and blood impurities, among others (Mahmood et al., 2010; Hamza, 2010). In addition to its traditional use as a vegetable, Moringa is recognized for its therapeutic properties, which include antipyretic, anti-ulcer, anti-epileptic, diuretic, cholesterol-lowering, renal protective, anti-diabetic, and hepatoprotective activities (Abalaka et al., 2009; Guevara et al., 1999).

**Figure 1:**
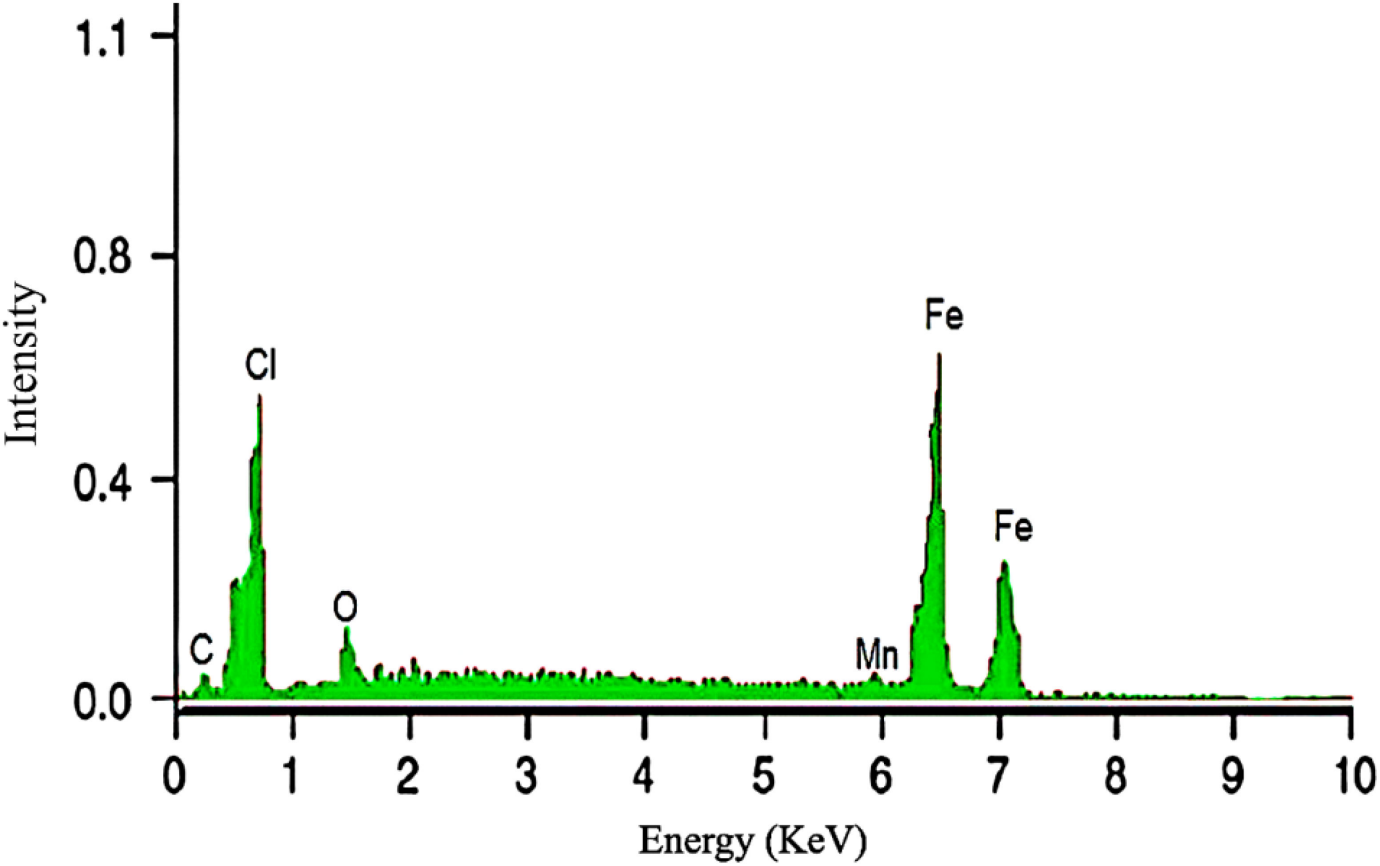
EDX of green synthesized IONPs.

The plant’s bioactive compounds are diverse and include alkaloids such as quinine, saponins, flavonoids, tannins, steroids, glycosides, niazinins, and niaziminins (**Figure 1**). These secondary metabolites contribute to *M. oleifera’s* potential as a green resource for the synthesis of nanoparticles (NPs) with various biological activities. Research has shown that compounds like flavonoids, phenolics, polysaccharides, and terpenoids, found in the leaves, flowers, stems, peel, and pods, play a critical role in the production of NPs with distinct sizes and shapes. These nanoparticles exhibit a range of notable biological properties, including antibacterial, antioxidant, cytotoxic, and anticancer effects (Aisida et al., 2019; Aliyu et al., 2017; El-Khadragy et al., 2018; Guevara et al., 1999; Shousha et al., 2019; Surendra et al., 2016; Vasanth et al., 2014). Iron is a crucial micronutrient for plant growth, playing a key role in chlorophyll biosynthesis, enzyme activation, and metabolic pathways. Conventionally, ferrous sulfate is used as an iron source in plant tissue culture media. However, its bioavailability is limited due to rapid oxidation, leading to the formation of insoluble ferric hydroxides that reduce nutrient absorption efficiency (Rout and Sahoo, 2015). Additionally, ferrous sulfate is prone to leaching, causing nutrient loss and environmental concerns. To address these limitations, iron oxide nanoparticles (IONPs) offer a novel and efficient alternative due to their higher stability, controlled release, and enhanced bioavailability in plant tissue culture systems. IONPs exhibit a sustained iron supply, reducing the risk of nutrient deficiencies and oxidative stress, which can hinder plant development. Furthermore, their nano-scale properties facilitate better uptake and transport within plant tissues, potentially improving overall growth, physiological responses, and secondary metabolite accumulation (Shirsat, 2024); (Khan et al., 2020).

Despite the recognized benefits of iron oxide nanoparticles (IONPs) in agriculture, their application as an iron source in *Stevia rebaudiana* micropropagation remains unexplored. This study introduces a novel approach by utilizing Moringa oleifera leaf extract for the green synthesis of IONPs and incorporating them into Murashige and Skoog (MS) medium as a replacement for ferrous sulfate. Unlike previous studies that focused on conventional elicitors to enhance steviol glycoside production, this research examines how IONPs influence micropropagation, antioxidant activity, and secondary metabolite accumulation in Stevia. The findings provide insights into dose-dependent effects, identifying optimal IONP concentrations that promote growth and the synthesis of bioactive compounds, including diterpene glycosides, in micropropagated Stevia plants while minimizing toxicity risks. By demonstrating the eco-friendly synthesis and agricultural potential of IONPs, this study paves the way for sustainable nanotechnology applications in plant tissue culture, enhancing crop growth and facilitating the production of medicinal metabolites for pharmaceutical industries.

## Methodology

### Preparation of plant extract and synthesis of iron oxide nanoparticles

A green synthesis approach was applied to synthesise IONPs in the Aquatic Plant Physiology Lab at the Institute of Hydrobiology, Chinese Academy of Sciences, Hubei Province. Iron sulphate (FeSO_4_) was chosen as the precursor salt due to its common use in nanoparticle formation. The IONPs were developed using an ethanolic extract of fresh, healthy leaves from *Moringa oleifera*, a heterophyllous aquatic plant.

Fresh leaves of *Moringa oleifera* were harvested from vegetatively propagated plants cultivated under controlled conditions. Only healthy, disease-free leaves were used to ensure the extract’s quality and consistency. The collected leaves were thoroughly washed with distilled water to remove any dirt or contaminants. After washing, the leaves were sliced into small pieces (approximately 10-20 g) and air-dried at room temperature for 24 hours to reduce moisture content.

The dried leaves were then used to prepare an ethanolic extract by mixing the plant material with 2:8 (*v/v*) ethanol to distilled water. To ensure that all phytochemicals were extracted, the mixture was agitated at room temperature for 24 hours. Following extraction, the mixture was filtered using Whatman No. 1 filter paper to remove any solid debris, leaving a clear, greenish solution. This filtered extract was maintained in a sterile container at 4°C in a refrigerator for future use. All experiments were carried out under sterile conditions to avoid contamination and retain the extract’s purity.

IONPs were synthesised by adding an ethanolic leaf extract to an aqueous FeSO_4_ solution (Shabbir et al., 2023). The leaf extract served as a reducing agent and stabiliser for nanoparticle production. The mixture was continuously stirred at room temperature in the dark for 2–3 hours. As the reaction progressed, the color of the solution changed, indicating the formation of iron oxide nanoparticles. After allowing the reaction to proceed for 24 hours, the solution was left undisturbed to ensure complete nanoparticle formation.

Following synthesis, the IONPs were isolated from the reaction solution by centrifugation at 10,000 rpm for 10 minutes. The supernatant was removed, and the pellet containing the nanoparticles was thoroughly rinsed with distilled water to eliminate any remaining chemicals or byproducts. The purified IONPs were resuspended in distilled water and analysed to validate their production as well as their morphological and structural features.

### Characterization of iron nanoparticles

#### Scanning electron microscopy

The morphology of biosynthesised iron nanoparticles was examined using scanning electron microscopy (SEM). The SEM imaging was carried out using a TESCAN MIRA3 model (TESCAN, Czech Republic), which has advance electron optics for high-resolution imaging. A small amount of nanoparticle suspension was drop-cast onto a carbon-coated copper grid, which was then air-dried. The grid was put in the SEM and imaged at various magnifications to determine the size, shape, and distribution of the nanoparticles.

#### Energy-dispersive x-ray spectroscopy

The elemental composition of the synthesised iron nanoparticles was determined using energy-dispersive X-ray spectroscopy (EDX). The EDX analysis was performed using a TESCAN MIRA3 SEM (TESCAN, Czech Republic), which had an integrated EDX detector. A little amount of nanoparticle sample was drop-coated onto a carbon film and let to dry. The coated sample was then subjected to an EDX detector, which produced a comprehensive spectrum of the elements contained in the nanoparticles. This examination validated the presence of iron (Fe) in the nanoparticle structure and enabled the detection of any potential contaminants or impurities.

### Preparation of media for micropropagation of *S. rebaudiana*

To assess the effects of IONPs on the growth, development, antioxidant profile, and stevioside accumulation in micropropagated *S. rebaudiana*, five different medium formulations were developed (**Table 1**). Micropropagation was carried out adopting the protocol established by Khan et al (Khan et al., 2020), with slight modifications matched to our experimental conditions. Sterile nodal segments of *S. rebaudiana* were employed as explants in the *in vitro* culture.

**Table 1:** Composition of MS media supplemented with iron sources and nanoparticles.

Treatment	Culture Media Composition
Positive Control	MS media supplemented with ferrous sulfate as the iron source
Negative Control	MS media without ferrous sulfate
5.60 mg/L IONPs	MS media without ferrous sulfate, supplemented with 5.60 mg/L IONPs
11.20 mg/L IONPs	MS media without ferrous sulfate, supplemented with 11.20 mg/L IONPs
22.40 mg/L IONPs	MS media without ferrous sulfate, supplemented with 22.40 mg/L IONPs

The MS (Murashige and Skoog) medium was prepared by dissolving 30 g/L sucrose, 3 mg/L benzylaminopurine (BA), and 7 g/L agar in distilled water. The pH of the medium was adjusted to 5.80 with a dilute solution of hydrochloric acid (HCl) or sodium hydroxide (NaOH). After complete mixing, the medium was autoclaved at 121°C for 15 minutes to remove any microbial contaminants.

After sterilisation and cooling to room temperature, IONPs suspensions with concentrations of 5.60 mg/L, 11.20 mg/L, and 22.40 mg/L were added to the medium. The IONP suspensions were made under sterile conditions and filtered through a 0.22 µm microfilter to eliminate aggregates and ensure uniform dispersion. To prevent nanoparticle aggregation, the suspension was gently added to the cool MS medium. To ensure a uniform distribution of IONPs, the final medium was poured into sterile test tubes (6 ± 1 mL each) and gently mixed by hand. The tubes were then placed in a refrigerator at 4°C to solidify the medium.

Sterile nodal explants were placed in prepared culture tubes and maintained under controlled environmental conditions for four weeks. During this period, data on morphological characteristics, including shoot and root initiation time, shoot and root count, and leaf number, were collected at regular intervals. The fresh and dry weights of the plantlets were also measured to assess overall growth. To determine the dry weight, the plantlets were carefully removed from the culture tubes, rinsed with distilled water, and dried at 50°C for two days until a constant weight was achieved.

#### Determination of total phenolic content

The total phenolic content (TPC) was determined using the previously reported method (Ali et al., 2019). A 20 μL sample (10 mg/mL) was put to a 96-well microplate, followed by 90 μL of 10X diluted Folin-Ciocalteu reagent. After 5-minute incubation at room temperature, 90 μL of sodium carbonate (20%) was added to make a final volume of 200 μL. A calibration curve was constructed with gallic acid (1-10 mg/mL) as a positive control and methanol (20 μL) as the negative control. After 90 minutes of incubation, the absorbance was measured at 630 nm with a Biotek microplate reader (ELX 800, BIOTEK). The results were represented in milligrams of gallic acid equivalents (GAE) per gram.

### Assessment of antioxidant activity using the DPPH assay

The antioxidant capacity of *S. rebaudiana* plantlets micropropagated under various treatments (positive control, negative control, and 5.60, 11.20, and 22.40 mg/L IONPs concentrations) was assessed using the DPPH (2,2-diphenyl-1-picrylhydrazyl) radical scavenging assay (Abbasi et al., 2010). For sample preparation, 1 mg of plant material from each treatment group was dissolved in 1 mL of dimethyl sulfoxide (DMSO) to make a stock solution. This stock was serially diluted to reach concentrations ranging from 25 to 100 μL. After each dilution, 50 μL was aliquoted into Eppendorf tubes, followed by 950 μL methanol. The tubes were then filled with 50 μL of DPPH solution (0.004%) for a total reaction volume of 1000 μL.

Ascorbic acid was used as a positive control, and DMSO as a negative control. The reaction mixture was incubated in dark at room temperature (25°C) for 30 minutes. The colour shift from purple to yellow was visually observed, with yellow signifying antioxidant activity in the sample. The absorbance of the reaction mixtures was determined spectrophotometrically at 517 nm using a UV-Vis spectrophotometer. The antioxidant activity was represented as a percentage of radical scavenging and calculated using the following formula:


$$\text{Antioxidant Activity }\left(\%\right)=\left(A_{control}–A_{sample}/A_{control}\right)\times 100$$

#### Determination of total flavonoid content

The total flavonoid content (TFC) was determined by adopting the protocol described by Khan et al (Khan et al., 2020). A 96-well microplate was filled with 20 μL of a 10 mg/mL sample, 10 μL of aluminium chloride (10 g/L), and 10 μL of potassium acetate (98.15 g/L). The negative and positive controls were methanol (20 μL) and quercetin (1.0 mg/mL), respectively. The final volume was adjusted to 200 μL with distilled water and incubated for 30 minutes. A standard curve was created with rutin (1-10 mg/mL). The absorbance was measured at 450 nm, and the findings were presented as milligrams of quercetin equivalents (QE) per gram.

#### Antioxidant enzyme activity assays

Antioxidant enzyme activities were evaluated using the method described by Ali et al. (2018). Samples were homogenised in 2 mL of potassium phosphate buffer (pH 7.8) containing 1% polyvinylpyrrolidone (PVP) and 0.1 mM ethylenediaminetetraacetic acid (EDTA) before centrifugation at 12,000 rpm for 15 minutes at 4°C. The supernatant was used to evaluate the activity of superoxide dismutase (SOD) (Giannopolitis and Ries, 1977). A 3 mL reaction mixture containing 63 mM nitro blue tetrazolium chloride (NBT), 13 mM methionine, 50 mM phosphate buffer, 1.4 mM riboflavin, and 50 µL of enzyme extract was incubated for 15 minutes, after which the absorbance was measured at 560 nm. For peroxidase (POD) activity, (Abeles and Biles, 1991) protocol was following and the oxidation of guaiacol in the presence of hydrogen peroxide (H_2_O_2_) was measured at 470 nm. To determine catalase (CAT) activity, 3 mL of the reaction mixture (50 mM PBS, 15 mM H_2_O_2_) was mixed with 50 mL of enzyme extract. The reaction was started by the addition of 100 μL of extract; an absorption decrease in H_2_O_2_ was measured at 240 nm (Arrigoni et al., 1992). The enzyme activity of ascorbate peroxidase (APX) was determined based on the oxidation of ascorbic acid, with absorbance measured at 290 nm. using a UV-Vis spectrophotometer (Miyake et al., 2006).

#### HPLC quantification of Stevioside and Rebaudioside A

Stevioside and Rebaudioside A were quantified using HPLC, as described by Kazmi et al (Kazmi et al., 2019b). Dried plant samples were extracted with a water-methanol mixture (1:9 *v/v*) at 30°C overnight. The extract was filtered, centrifuged, concentrated by vacuum, re-filtered (0.45 μm), and transferred to HPLC vials. Reference standards of stevioside and rebaudioside A (Sigma-Aldrich, USA) were lyophilized and dissolved in HPLC-grade MiliQ water. HPLC analysis was carried out using an Agilent 100 HPLC system with a Luna C_18_ column (250 mm × 4.6 mm, 5 μm). The mobile phases were acetonitrile/water (85:15 *v/v*) for phase A and acetonitrile/water (75:25 *v/v*) for phase B, each with a flow rate of 1 mL/min. Detection was performed at 210 nm, with spectra ranging from 200 to 700 nm. Stevioside and rebaudioside A were identified based on their retention times compared to reference standards.

### Statistical analysis

Statistical analyses for all the selected parameters were conducted using Statistix version 8.1 to ensure the reliability and accuracy of the results. One-way analysis of variance (ANOVA) was applied to evaluate significant differences among different treatment groups. To further identify specific group differences, Tukey’s *post-hoc* test was performed, with a significance threshold set at p ≤ 0.05. This approach allowed for multiple comparisons while minimizing the risk of type I errors. Additionally, all experimental data were graphically represented using GraphPad Prism 5, which facilitated clear visualization and interpretation of trends across different parameters.

## Results and discussion

### Leaf extract of *Moringa oleifera* proficiently synthesized IONPs

**Figure 2:**
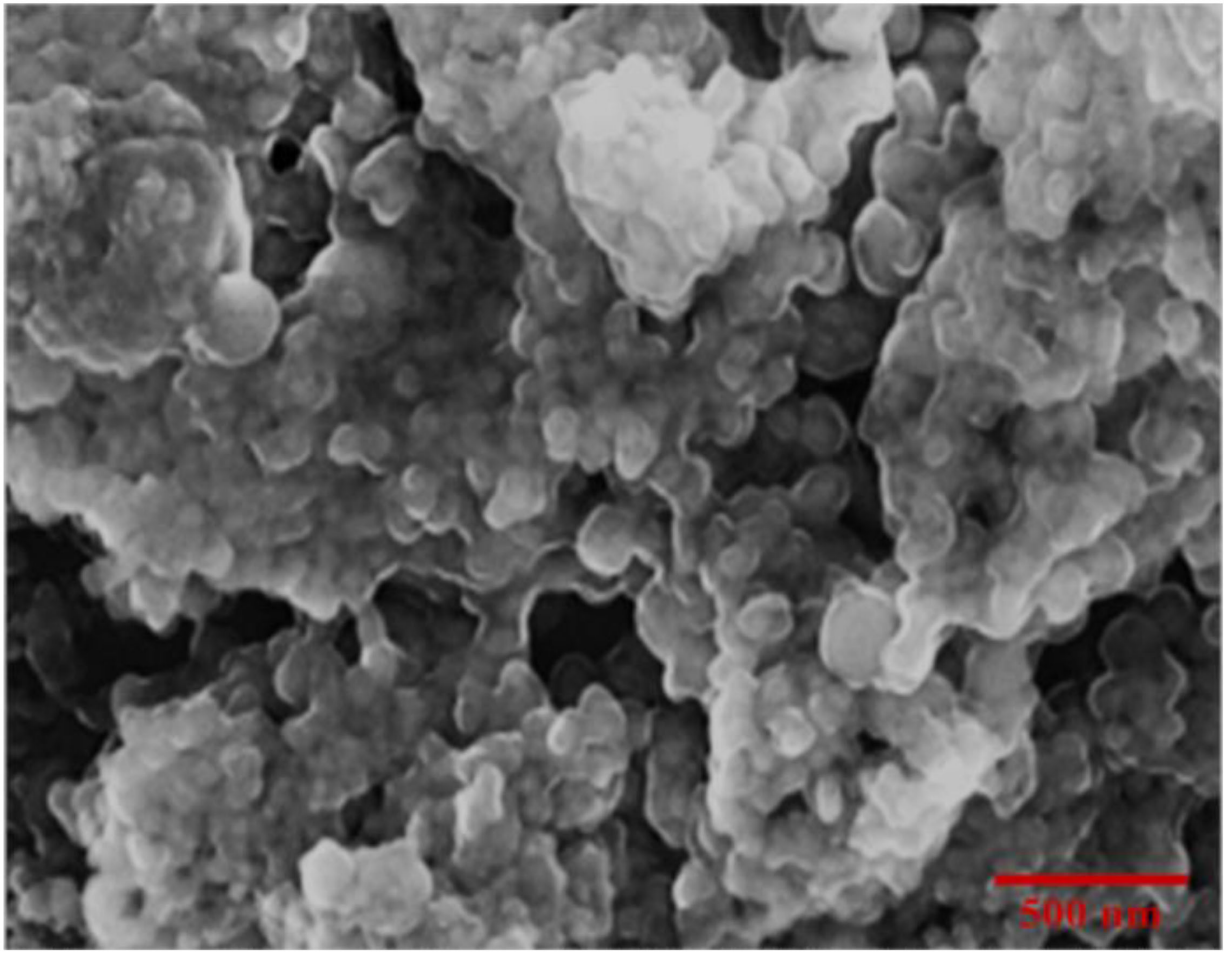
Scanning electron micrograph of phytosynthesized IONPs.

The application of cost-effective, sustainable, and environment friendly nanotechnology is an appealing approach of increasing agricultural productivity (Wahab et al., 2024; Shereen et al., 2024). Green-synthesized IONPs have received significant attention for their ability to enhance plant growth and development, making them an important tool to improve agricultural systems (Selvaraj et al., 2022; Dowlath et al., 2021). Plant extracts play an important role in the synthesis of these nanoparticles as their active compounds facilitate the reduction of iron ions to nanoparticles (Bouafia and Laouini, 2021; Ikhuoria et al., 2024; Saod et al., 2024; Duan et al., 2024). Despite its traditional medicinal uses, the potential of Moringa oleifera mediated IONPs application in *Stevia rebaudiana in vitro* plantlet development remains unexplored. Notably, no published research has reported the synthesis of iron oxide nanoparticles (IONPs) using *Moringa oleifera* leaf extract, and their replacement with ferrus sulfate in stevia micropropagation, a gap this study aims to address. *Moringa oleifera* was selected for the green synthesis of IONPs due to its rich phytochemical profile, which plays a crucial role in nanoparticle reduction and stabilization (Kiwumulo et al., 2022). The plant is known for its high content of polyphenols, flavonoids, alkaloids, tannins, and proteins, which act as natural capping and stabilizing agents, enabling efficient nanoparticle synthesis without the need for toxic chemical reagents (Yadav et al., 2024). Additionally, *Moringa oleifera* has been extensively studied for its antioxidant, antimicrobial, and biocompatible properties, making it a promising candidate for eco-friendly nanomaterial synthesis. Its abundant availability, cost-effectiveness, and sustainability further support its selection as a green reducing agent for nanoparticle fabrication (Ahmed et al., 2022). The bioactive compounds in Moringa not only aid in the synthesis of stable IONPs but may also enhance their biological activity, improving their effectiveness in plant tissue culture applications.By utilizing *Moringa oleifera* for IONP synthesis, this study aligns with the principles of green chemistry, offering an eco-friendly, non-toxic, and sustainable approach to developing nanoparticles with enhanced biocompatibility and bioactivity (Angle, 2024). In the present study, *Moringa oleifera* leaf extract was used to produce IONPs, which were subsequently added into Murashige and Skoog (MS) medium to replace the conventional iron source. The goal was to assess the effects of these nanoparticles on *S. rebaudiana* micropropagation, with an emphasis on antioxidant potential, antioxidant enzyme activity, and secondary metabolite accumulation, including phenolics, flavonoids, and diterpene glycosides. At room temperature, IONPs of adequate size and shape were synthesised. SEM and EDX analysis revealed spherical or polygonal IONPs (**Figure 2**), with the highest iron peak detected in the 6.5-7.5 keV range (**Figure 1**), which supports previous findings (Elemike et al., 2023; Samuel et al., 2021). This eco-friendly synthesis process reduces toxicity and offers a sustainable option for nanoparticle production.

### IONPs promoted micropropagation, leaf development, polyphenols production in of *S. rebaudiana*

**Figure 3:**
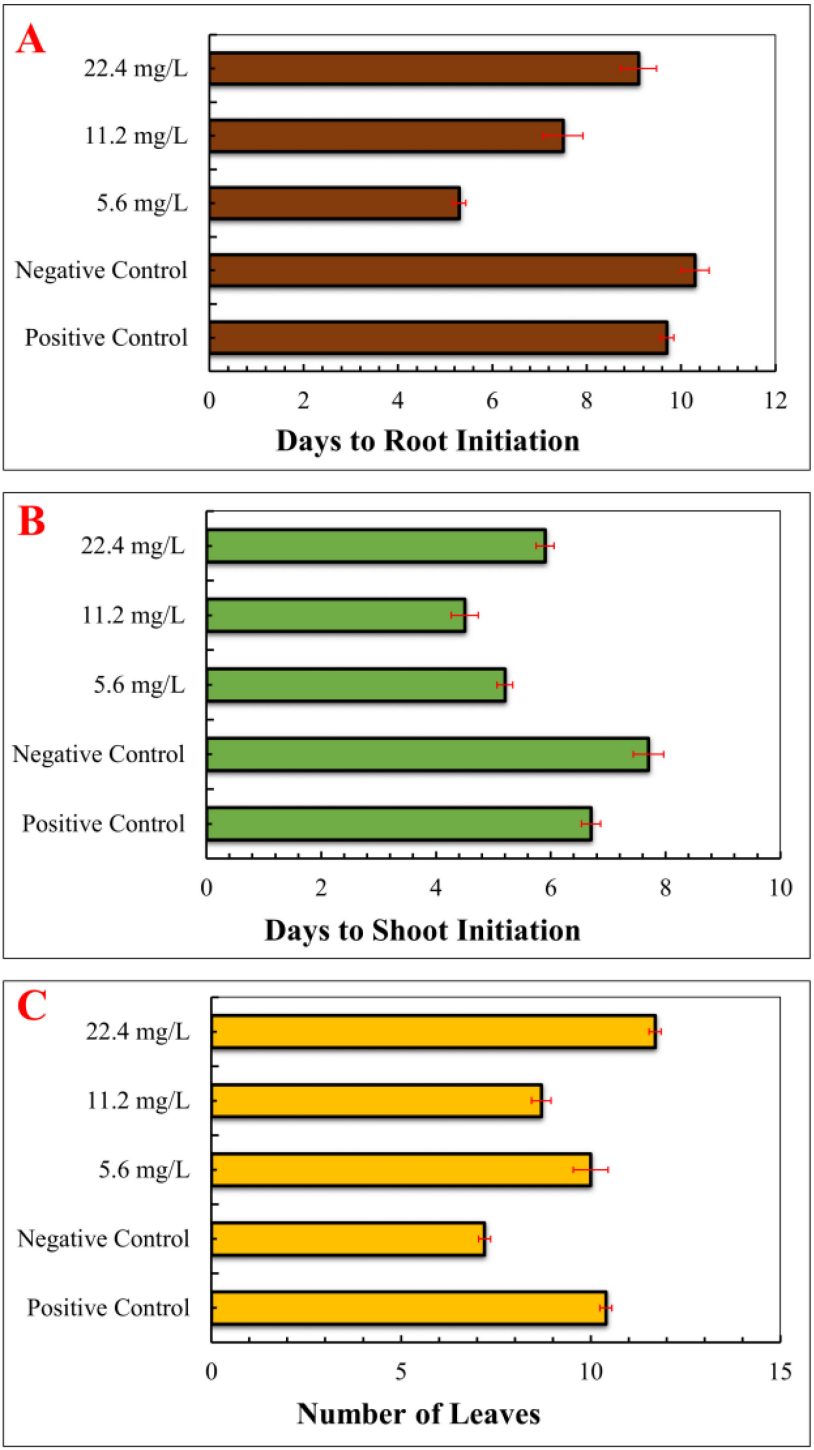
Effect of various concentrations of IONPs on the micropropagation of *S. rebaudiana*. **(A)** Days to root initiation measured under different treatments: Positive Control (MS medium with ferrous sulfate), Negative Control (MS medium without ferrous sulfate), and iron oxide nanoparticle (IONP)-supplemented treatments (5.6 mg/L, 11.2 mg/L, and 22.4 mg/L IONPs). **(B)** Days to shoot initiation of plantlets grown under the same conditions. **(C)** Number of leaves. Error bars represent standard deviation (SD) from triplicate measurements. Different letters indicate statistically significant differences between treatments (p ≤ 0.05, Tukey’s *post-hoc* test, one-way ANOVA).

The effects of IONPs on morphology, antioxidant defence system, and stevioside synthesis in *S. rebaudiana* were investigated. Our results indicate that the effect of IONPs on *S. rebaudiana* growth is dose-dependent. Plants grown on MS basal medium containing Fe²^+^ (as FeSO_4_·7H_2_O) exhibited normal growth, as did plants grown on MS media supplemented with various concentrations of FeNPs. Notably, lower FeNP concentrations (5.6 mg/L) promoted early shoot and root initiation, with shoots appearing after 5.2 days and roots after 5.3 days. Higher IONP concentrations (11.20 and 22.40 mg/L) resulted in delayed shoot and root initiation (7.5 and 9.1 days, respectively) (**Figure 3**). These findings indicate that excessive IONPs may cause toxicity, affecting plant growth and development, which is consistent with finding of similar effects on other plant species.

Overall, lower concentrations of IONPs aided plant development, though higher concentrations considerably inhibited growth parameters. These results are consistent with the findings of Khan et al (Khan et al., 2020), who reported that early root and shoot initiation was promoted by lower FeNP concentrations (45 µg/L) and delayed by higher concentrations (90 and 135 µg/L). According to Jamzad Fard et al (Jamzad Fard et al., 2013), *Rosa chinensis* developed better when exposed to ideal FeNP concentrations *in vitro*. Similarly, Sheykhbaglou et al. (2010) and Rui et al. (2016) reported that IONPs at low concentrations boosted soybean and peanut biomass, respectively, however higher doses had detrimental effect. In a recently published study IONPs considerably inhibited growth at high concentrations but had a favourable effect on C*arum copticum* L. at low amount (Razavizadeh et al., 2024). Numerous scientific studies have reported that various nanoparticles can have both positive and negative effects on plant growth and productivity. While some nanoparticles enhance plant development, others may lead to growth inhibition and reduced yield, depending on their concentration and exposure conditions (Ali et al., 2019; Khan et al., 2019b; Ma et al., 2016; Mohammad et al., 2019; Sarmast et al., 2015).

**Figure 4:**
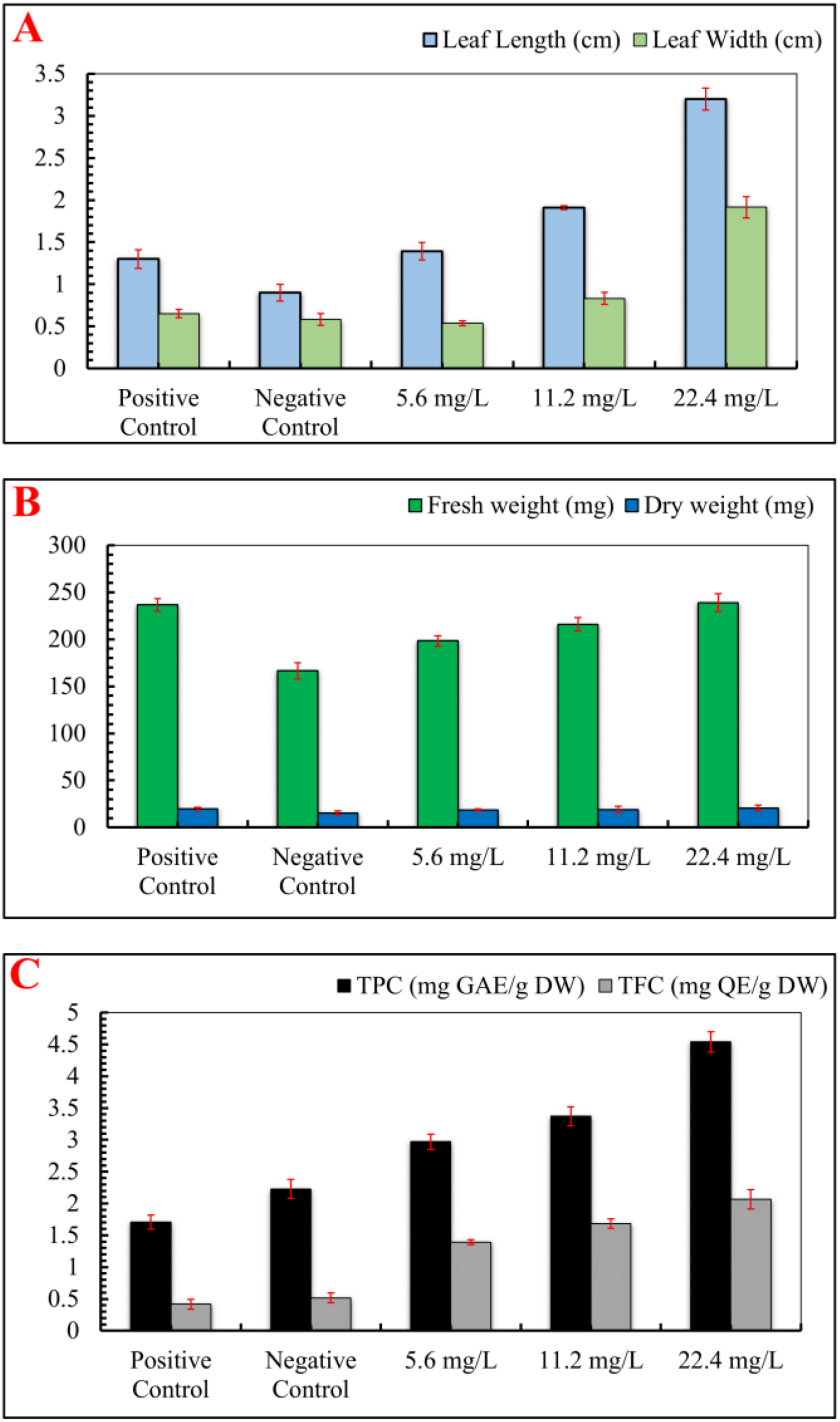
Effect of various concentrations of IONPs on the leaf size, biomass and phytochemicals of micropropagated *S. rebaudiana*. **(A)** Leaf length (cm) and leaf width (cm) measured under different treatments: Positive Control (MS medium with ferrous sulfate), Negative Control (MS medium without ferrous sulfate), and iron oxide nanoparticle (IONP)-supplemented treatments (5.6 mg/L, 11.2 mg/L, and 22.4 mg/L IONPs). **(B)** Fresh weight (mg) and dry weight (mg) of plantlets grown under the same conditions. **(C)** Total phenolic content (TPC) expressed as mg gallic acid equivalent per gram of dry weight (mg GAE/g DW) and total flavonoid content (TFC) expressed as mg quercetin equivalent per gram of dry weight (mg QE/g DW). Error bars represent standard deviation (SD) from triplicate measurements. Different letters indicate statistically significant differences between treatments (p ≤ 0.05, Tukey’s *post-hoc* test, one-way ANOVA).

Interestingly, IONPs improved various growth parameters in *S. rebaudiana*, including leaf length, leaf width, number of leaves, fresh weight, and dry weight. The IONPs treatment at 22.4 mg/L increased leaf width (1.92 cm), length (3.20 cm), number of leaves (n = 11.70), fresh weight (238.90 mg), and dry weight (20.67 mg) in comparison to the positive control (FeSO_4_·7H_2_O) and other treatments (**Figure 4**). IONPs effectively replaced FeSO_4_·7H_2_O, increasing leaf length, width, fresh and dry weight to levels comparable to the control. These results are supported by Desai Heta et al (Desai Heta et al., 2017), who reported that magnesium nanoparticles (MgNPs) increased the length and width of *S. rebaudiana* leaves. IONPs have also been shown to have positive impacts on plant development in date palm (Arafa et al., 2023), *Cicer arietinum* (Irum et al., 2020), and *Oryza sativa* (Ullah et al., 2024).

Subsequent analysis revealed that IONPs increased the concentrations of flavonoids and phenolic compounds in *S. rebaudiana*. In comparison to the positive control (1.71 mg GAE/g and 0.42 mg QAE/g), the total phenolic content (TPC) and total flavonoid content (TFC) were maximum at 22.40 mg/L FeNPs (4.54 mg GAE/g and 2.07 mg GAE/g, respectively). Lower IONPs concentrations (5.6 and 11.2 mg/L) also increased TPC and TFC, though moderately. Our findings correlate with the previous studies, which found enhanced phenolic and flavonoid content in response to nanoparticles (Rastegaran et al., 2022, Sahraei et al., 2023; Khanizadeh et al., 2024; Ayoobi et al., 2024; Khan et al., 2021). Previous studies utilizing different nanoparticles on *in vitro* cultures of *Stevia rebaudiana* have demonstrated significant enhancements in phenolic and flavonoid content (Ghazal et al., 2018; Golkar et al., 2019; Javed et al., 2017a). While iron oxide nanoparticles (IONPs) serve as a potential alternative to conventional iron supplements in plant tissue culture, their effects are highly concentration-dependent. At optimal concentrations, IONPs enhance plant growth, biomass accumulation, and secondary metabolite production by facilitating iron uptake and promoting physiological activities (Irum et al., 2020). However, at higher concentrations (e.g., 22.40 mg/L IONPs), phytotoxic effects become evident, leading to growth inhibition, oxidative stress, and metabolic disturbances. One of the primary mechanisms of IONP-induced phytotoxicity is the generation of reactive oxygen species (ROS), which can result in lipid peroxidation, protein oxidation, and DNA damage. Excessive ROS levels disrupt cellular homeostasis, impairing photosynthesis, enzymatic activity, and nutrient absorption (Khan et al., 2023). In the current study, higher IONP concentrations negatively impacted leaf morphology, biomass accumulation, and antioxidant enzyme activities, indicating oxidative stress and cellular toxicity. Moreover, excessive IONP exposure can lead to iron overload, causing iron-induced Fenton reactions, which further exacerbate oxidative damage. This can alter chlorophyll biosynthesis, reduce photosynthetic efficiency, and impair root and shoot development (Onaga et al., 2016). The study’s findings suggest that while moderate IONP supplementation (5.60-11.20 mg/L) promotes plant growth, exceeding this threshold results in detrimental effects on overall plant health and metabolic stability. Thus, careful dose optimization is crucial to harness the benefits of IONPs while minimizing their phytotoxic effects. Future studies should explore mechanisms of nanoparticle uptake, accumulation, and detoxification pathways to mitigate toxicity risks and enhance the application of nanotechnology in plant culture systems.

### IONPs significantly enhanced antioxidant potential and diterpene glycosides accumulation in micro-propagated plantlets of *S. rebaudiana*

**Figure 5:**
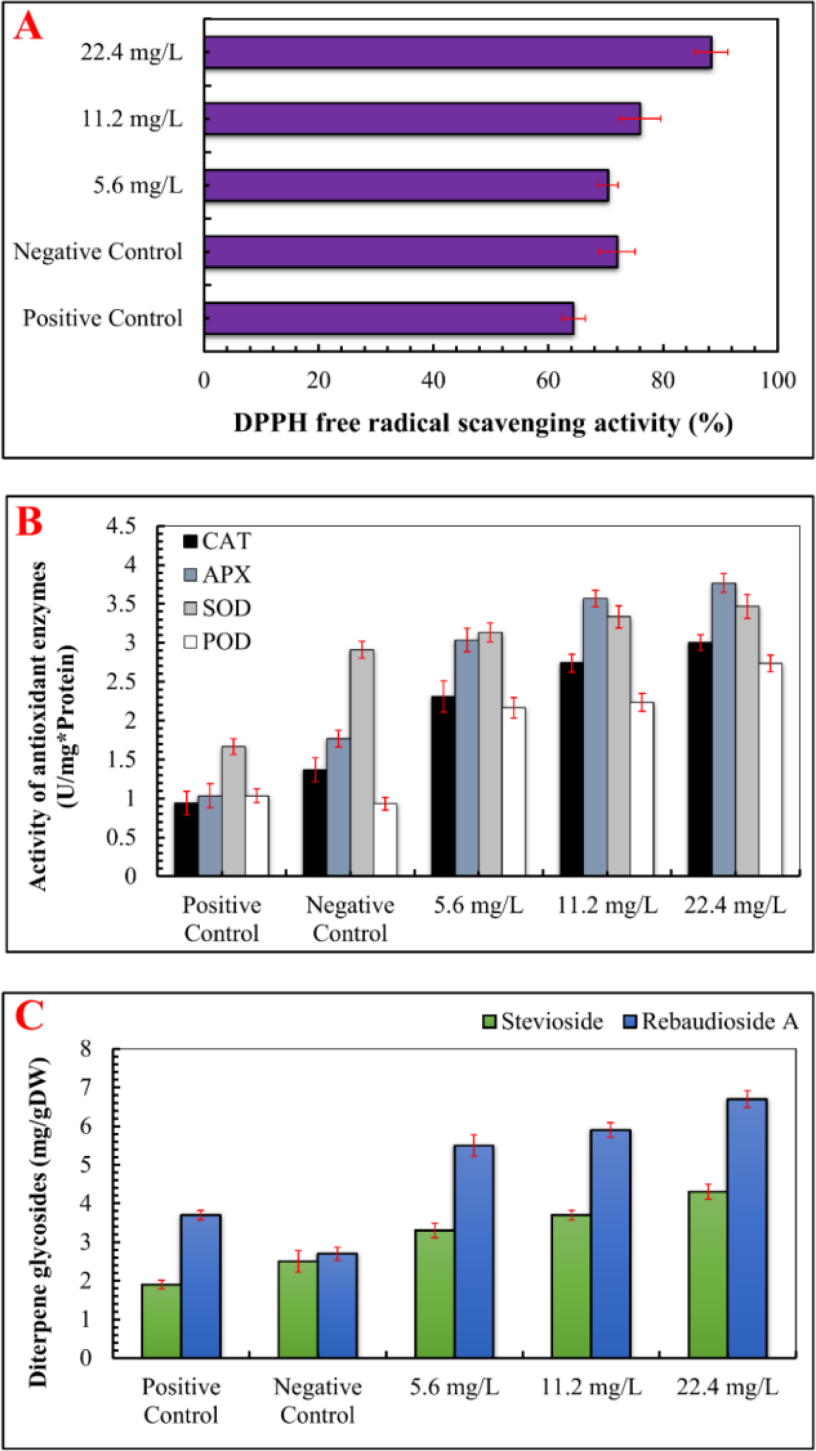
Effect of various concentrations of IONPs on the antioxidant profile and accumulation of secondary metabolites in micropropagated *S. rebaudiana*. **(A)** DPPH free radical scavenging activity (%) measured under different treatments: Positive Control (MS medium with ferrous sulfate), Negative Control (MS medium without ferrous sulfate), and iron oxide nanoparticle (IONP)-supplemented treatments (5.6 mg/L, 11.2 mg/L, and 22.4 mg/L IONPs). **(B)** B) Activity of antioxidant enzymes i.e SOD, POD, CAT, APx (U mg*/Protien) of plantlets grown under the same conditions. **(C)** Concentration of diterpene glycosides expressed as mg per gram of dry weight (mg/g DW). Error bars represent standard deviation (SD) from triplicate measurements. Different letters indicate statistically significant differences between treatments (p ≤ 0.05, Tukey’s *post-hoc* test, one-way ANOVA).

When exposed to abiotic stress, plants generate reactive oxygen species (ROS), which disrupt normal physiological functions. To counteract this, plants produce enzymes that neutralize ROS (Wang et al., 2024; Fan and Li, 2024). In the present study, DPPH free radical scavenging activity (%) was assessed, and it was observed that IONP treatment significantly enhanced free radical scavenging activity (89.70%). At lower IONP concentrations (5.60 and 11.20 mg/L), DPPH activity showed a moderate increase (**Figure 5**). Iron oxide nanoparticles are known to facilitate iron release in plant cells and stimulate the Fenton reaction, ultimately leading to the generation of reactive free radicals (Taghizadeh et al., 2019). Similarly, the activity of ROS-scavenging enzymes, such as catalase (CAT), ascorbate peroxidase (APX), peroxidase (POD), and superoxide dismutase (SOD) was also increased as compared to the negative control. Compared to the Fe-deficient plants (1.77 U/mg protein), the maximum activity of APX (3.77 U/mg protein) was detected at 22.40 mg/L IONPs. Similarly, the maximum CAT activity (3 U/mg protein) was detected at 22.4 mg/L IONPs, followed by considerable increases in SOD (3.46 U/mg protein) and POD (2.73 U/mg protein) activities.

**Figure 6:**
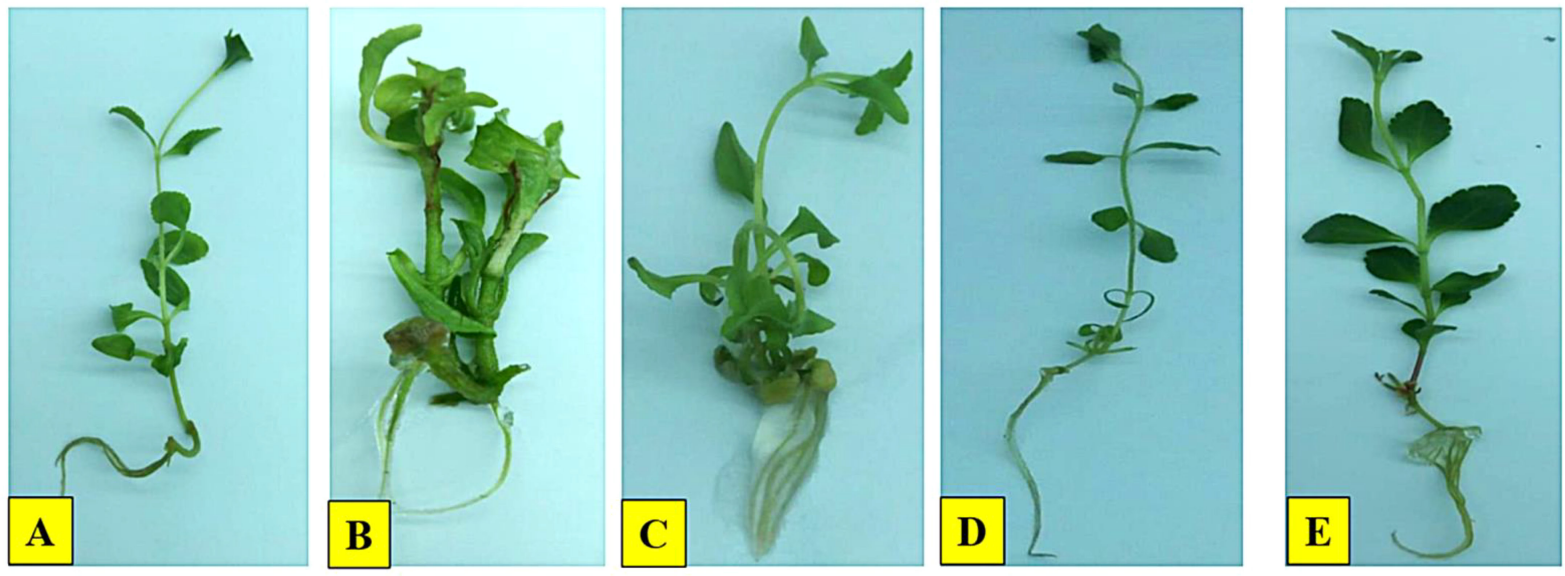
Effect of IONPs on micropropagation of *S. rebaudiana*. **(A)** Positive control, **(B)** Negative control, **(C)** 5.60 mg/L, **(D)** 11.20 mg/L, **(E)** 22.40 mg/L.

**Figure 7:**
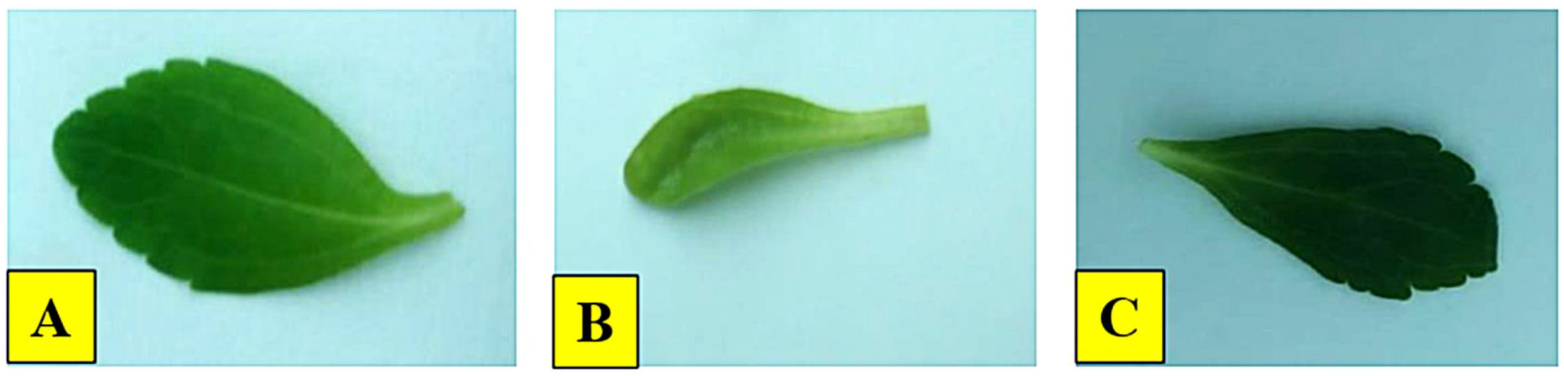
Effect of IONPs on the leaves of micropropagated *S. rebaudiana*. **(A)** 22.4 mg/L, **(B)** Negative control, **(C)** Positive control.

ROS-induced oxidative damage might considerably impair macromolecules, eventually leading to cell death (Demidchik, 2015). ROS can degrade chlorophyll content and activate secondary metabolic pathways as a stress response (Yasmeen et al., 2024). Previous studies have shown that metal nanoparticles promote the formation of ROS, which triggers secondary metabolism and reduces plant damage (Hatami and Ghorbanpour, 2024; Prasad et al., 2024; Selvakesavan et al., 2023; Lala, 2021). Furthermore, our results revealed that maximum levels of stevioside (4.30 mg/g DW) and Rebaudioside A (6.70 mg/g DW) accumulation were promoted by IONPs at higher concentration (22.40 mg/L). A moderate level of increase in diterpene glycosides (Stevioside: 3.3 mg/g DW, Rebaudioside A: 5.5 mg/g DW) was also detected in plants micrpropagated under lower IONP concentrations (5.60 and 11.20 mg/L). The increase in steviol glycoside production can be attributed to improved leaf health, as these compounds are synthesized in chloroplasts (**Figures 6**, **7**). Ensuring effective steviol glycoside synthesis and maintaining chloroplast integrity depend on optimal iron levels. Iron plays a crucial role in over 100 enzymes essential for various metabolic processes (Briat et al., 2007).

The enhancement of steviol glycosides in *Stevia rebaudiana* has been linked to the activation of glycosyltransferase enzymes, which are influenced by external stress-inducing compounds. These enzymes facilitate the transfer of sugar residues from donor molecules to acceptors, thereby promoting the biosynthesis of steviol glycosides (Vázquez-Hernández et al., 2019). Iron is another crucial factor for optimal plant growth and proper chloroplast development. Both deficiency and excess of iron can disrupt metabolic processes. Iron plays a key role in activating over 100 enzymes essential for biochemical reactions. Several studies have reported that various elicitors can significantly increase the production of stevioside and rebaudioside A in *S. rebaudiana* by upregulating enzymes involved in secondary metabolism (Kazmi et al., 2019a). For instance, Golkar et al. (2019) found that treating callus cultures of *S. rebaudiana* with 45 mg/L of silver nanoparticles (AgNPs) enhanced the accumulation of rebaudioside A and stevioside. Similarly, Javed et al. (2017b) investigated the effects of zinc oxide nanoparticles (ZnO NPs) on shoot regeneration and steviol glycoside production, reporting that a concentration of 1 mg/L ZnO NPs promoted optimal shoot formation and increased glycoside content. However, higher doses had adverse effects on growth and biosynthesis. Additionally, Magangana et al. (2018) examined the influence of nitrogen and phosphate on *S. rebaudiana* cultures and found that these nutrients had varying impacts on growth and stevioside biosynthesis.

## Conclusion

This study successfully demonstrates the green synthesis of iron oxide nanoparticles (IONPs) using *Moringa oleifera* leaf extract, which functioned as both a reducing and capping agent. The synthesized IONPs exhibited spherical and polygonal morphologies, and their integration into Murashige and Skoog (MS) medium as a substitute for conventional iron sources significantly influenced the micropropagation of Stevia rebaudiana. A dose-dependent response was observed, wherein lower IONP concentrations (5.60 mg/L) promoted early shoot and root initiation, whereas higher concentrations (11.20 mg/L and 22.40 mg/L) led to growth delays and inhibited development. At optimal concentrations, IONPs enhanced leaf morphology, increased phenolic and flavonoid content, improved antioxidant activity, and stimulated the accumulation of diterpene glycosides, particularly stevioside and rebaudioside A. These findings underscore the potential of IONPs as a sustainable and effective strategy for enhancing plant growth and secondary metabolite production in S. rebaudiana, offering valuable insights for agricultural and pharmaceutical applications.

## Future prospects

The promising effects of IONPs on plant growth and secondary metabolite biosynthesis warrant further investigation into the underlying mechanisms, particularly the molecular pathways regulating antioxidant defense and metabolic responses. Future research should focus on optimizing the concentration and application of IONPs across various crops to enhance agricultural productivity. Additionally, long-term studies assessing the environmental impact and potential bioaccumulation of IONPs in plant tissues are crucial to ensuring their safety and sustainability in agricultural systems. Such investigations could facilitate the broader application of nanotechnology in sustainable agriculture, providing innovative solutions to improve crop yield and quality.

## Data Availability

The raw data supporting the conclusions of this article will be made available by the authors, without undue reservation.
